# Diurnal variation in the proinflammatory activity of urban fine particulate matter (PM _2.5_) by *in vitro* assays

**DOI:** 10.12688/f1000research.14836.3

**Published:** 2018-10-01

**Authors:** Christopher Lovett, Mafalda Cacciottolo, Farimah Shirmohammadi, Amin Haghani, Todd E. Morgan, Constantinos Sioutas, Caleb E. Finch

**Affiliations:** 1 Department of Civil and Environmental Engineering, University of Southern California, Los Angeles, CA, 90089, USA; 2 Leonard Davis School of Gerontology, University of Southern California, Los Angeles, CA, 90089, USA

**Keywords:** Photochemistry, Los Angeles, PM2.5, Oxidative stress, Traffic, Primary PM, Secondary PM, Neuroinflammation

## Abstract

**Background:** Ambient particulate matter (PM) smaller than 2.5 µm in diameter (PM _2.5_) undergoes diurnal changes in chemical composition due to photochemical oxidation. In this study we examine the relationships between oxidative activity and inflammatory responses associated with these diurnal chemical changes. Because secondary PM contains a higher fraction of oxidized PM species, we hypothesized that PM _2.5_ collected during afternoon hours would induce a greater inflammatory response than primary, morning PM _2.5_.

**Methods:** Time-integrated aqueous slurry samples of ambient PM _2.5_ were collected using a direct aerosol-into-liquid collection system during defined morning and afternoon time periods. PM _2.5_ samples were collected for 5 weeks in the late summer (August-September) of 2016 at a central Los Angeles site. Morning samples, largely consisting of fresh primary traffic emissions (primary PM), were collected from 6-9am (am-PM _2.5_), and afternoon samples were collected from 12-4pm (pm-PM _2.5_), when PM composition is dominated by products of photochemical oxidation (secondary PM). The two diurnally phased PM _2.5_ slurries (am- and pm-PM _2.5_) were characterized for chemical composition and BV-2 microglia were assayed *in vitro* for oxidative and inflammatory gene responses.

**Results:** Contrary to expectations, the am-PM _2.5_ slurry had more proinflammatory activity than the pm-PM _2.5_ slurry as revealed by nitric oxide (NO) induction, as well as the upregulation of proinflammatory cytokines IL-1β, IL-6, and CCL2 (MCP-1), as assessed by messenger RNA production.

**Conclusions:** The diurnal differences observed in this study may be in part attributed to the greater content of transition metals and water-insoluble organic carbon (WIOC) of am-PM _2.5_ (primary PM) vs. pm-PM _2.5_ (secondary PM), as these two classes of compounds can increase PM _2.5_ toxicity.

## Introduction

Particulate matter (PM) with an aerodynamic diameter less than 2.5 µm (fine PM or PM _2.5_), is associated with diverse health problems and chronic diseases, including asthma, chronic obstructive pulmonary disease (COPD), lung cancer, and coronary heart disease ( Delfino *et al.*, 2005; Delfino *et al.*, 2011; Dockery *et al.*, 1993; Dominici *et al.*, 2006; Kaufman *et al.*, 2016; Kim *et al.*, 2013; Landrigan *et al.*, 2018; Shah *et al.*, 2013). Findings of recent epidemiological studies extend chronic PM _2.5_ exposure risk to Alzheimer’s disease and accelerated cognitive decline ( Cacciottolo *et al.*, 2017; Chen *et al.*, 2015; Chen *et al.*, 2017). Corresponding rodent models show robust indicators of inflammatory and oxidative stress to PM _2.5_ fractions in pathological responses of aorta ( Li *et al.*, 2003), brain ( Cheng *et al.*, 2016b; Levesque *et al.*, 2011; MohanKumar *et al.*, 2008; Morgan *et al.*, 2011), and lung ( Zhang *et al.*, 2012).

In addition to the epidemiological associations with chronic disease, we must also consider diurnal variations in airborne particulate matter chemistry that are not included in most long-term epidemiological studies. Diurnal variation in air pollution toxicity is suggested by diurnal variations in emergency department admissions for dementia ( Linares *et al.*, 2017), ischemic stroke ( Han *et al.*, 2016), and respiratory conditions ( Darrow *et al.*, 2011). Although these admissions were more strongly associated with ozone than with PM _2.5_ in all three of these studies, diurnal changes in PM _2.5_ chemistry must also be considered as an influencing factor. Freshly emitted primary PM undergoes photochemical oxidation reactions over the course of the day, catalyzed by ultraviolet (UV) sunlight, which results in diverse oxidized organic and inorganic products (secondary PM) ( Forstner *et al.*, 1997; Grosjean & Seinfeld, 1989), along with concomitant changes in PM toxicity. These diurnal changes in PM _2.5_ composition and associated toxicity are relevant to and may inform future long-term epidemiological studies of primary and secondary particulate matter. While prior studies in the Los Angeles air basin have shown extensive diurnal variations in PM composition and size, the findings of PM oxidative activity have been inconsistent and differ between various assays of oxidative potential ( Saffari *et al.*, 2015; Verma *et al.*, 2009; Wang *et al.*, 2013b).

The current study further examined diurnal variations in composition and oxidative potential of PM samples collected at the central Los Angeles site used in the three studies mentioned above. However, unlike these earlier studies, PM samples were collected by a direct aerosol-into-liquid collection method to provide time-integrated aqueous PM _2.5_ slurries for both morning and afternoon periods. This technology allows for a more comprehensive analysis than the filterable (i.e. water extracted) particulate samples examined in our prior studies ( Morgan *et al.*, 2011; Saffari *et al.*, 2015; Verma *et al.*, 2009; Woodward *et al.*, 2017a).

Microglia were used for *in vitro* assays of oxidative and inflammatory responses to PM _2.5_ exposures because of their increasingly recognized role in environmental neurotoxicology ( Krafft, 2015). Air pollution can induce premature microglial activation, as documented in rodent models ( Cheng *et al.*, 2016a; Hanamsagar & Bilbo, 2017; Morgan *et al.*, 2011) and as indicated for young adults living in the highly polluted Mexico City ( Calderón-Garcidueñas *et al.*, 2008; Calderón-Garcidueñas *et al.*, 2018). Microglia (BV-2) cell cultures were assayed for induction of nitric oxide (NO) and for proinflammatory gene mRNA responses of interleukins 6 and 1β (IL-6 & IL-1β), and monocyte chemoattractant protein 1 (MCP-1), also known as chemokine (C-C motif) ligand 2 (CCL2). These markers were chosen because of their *in vivo* and *in vitro* responses to ultrafine PM shown in prior studies ( Cheng *et al.*, 2016b; Morgan *et al.*, 2011; Woodward *et al.*, 2017b).

We hypothesized that afternoon PM _2.5_ (pm-PM _2.5_), with its high proportion of secondary photochemical oxidation products, would have greater oxidative and proinflammatory activity than freshly emitted, primary PM collected during morning hours (am-PM _2.5_).

## Methods

### Particulate sample collection

All sampling was done at the University of Southern California Particle Instrumentation Unit (PIU), located approximately 150 meters downwind (east) of the Los Angeles I-110 freeway (34°1’9” N, 118°16’38” W). PM _2.5_ samples were collected weekdays during the morning rush hour period of 6am–9am, as well as during the afternoon hours of 12pm–4pm, when photochemical products of primary PM oxidation are dominant in the atmosphere. The 5-week sampling campaign was conducted during late summer (August and September) of 2016, ensuring maximum UV sunlight exposure to enhance photochemical oxidation reactions.

Particle collection employed a novel high-volume aerosol-into-liquid collector developed and built at USC’s Sioutas Aerosol Laboratory, which provides concentrated slurries of fine and/or ultrafine PM ( Wang *et al.*, 2013a). A 2.5 µm cut-point slit impactor at the inlet to the online sampling system removed PM larger than 2.5 µm in diameter and ensured that only PM _2.5_ was captured in the aerosol-into-liquid collector. This sampler operates at 200 liters per minute (lpm) flow; two inlet aerosol streams, each at 100 lpm flow, are merged and passed through a steam bath where ultrapure water vapor condenses on the surfaces of airborne particles, growing the droplets to 2–3 μm in diameter. Downstream of the hot water bath, particles enter an electronic chiller, where they are cooled and condensed, passing through an impactor and accumulating in the aerosol-into-liquid collector as an aqueous PM _2.5_ slurry.

For each sampling condition, morning and afternoon, one time-integrated slurry sample was collected for chemical speciation and biological assays. At the end of each morning and afternoon daily sampling period, each aqueous slurry sample was added to its corresponding total sample collection bottle that was kept refrigerated. At the end of the 5-week sampling period, these continuously refrigerated, cumulative aqueous slurry samples were then used in the *in vitro* assays. While it is possible that changes in PM composition might occur during sampling, the advantage of using the direct aerosol-into-liquid system is that PM is collected directly into an aqueous suspension and does not undergo an aqueous extraction and re-suspension process, thereby significantly reducing the possibility of any artifact formation. The benefits of this collection method compared to conventional filter sampling systems have been discussed extensively in the literature (e.g. Saarikoski *et al.*, 2014; Wang *et al.*, 2013b; Zhao *et al.*, 2005).

### PM gravimetric analysis

To determine mass loadings of the PM _2.5_ slurry samples, 47 mm Zefluor filters (Pall Life Sciences, Ann Arbor, MI, USA) were used to capture PM _2.5_ passing through a parallel airstream at a flow rate of 9 lpm. Mass of the PM _2.5_ filter samples was determined gravimetrically by pre- and post-weighing the Zefluor filters, equilibrated at controlled temperature (22–24 °C) and relative humidity (of 40–50%) conditions. Slurry PM concentrations were calculated from the filter mass loadings and air volume sampled per time period.

### PM chemical species analysis

Aqueous PM _2.5_ slurry samples were analyzed for metals and trace elements, total carbon (TC), and inorganic ions. Analyses were performed in triplicate on one aliquot of each slurry, morning (am-PM _2.5_) and afternoon (pm-PM _2.5_). Total metals and trace elements were quantified using magnetic-sectored Inductively Coupled Plasma Mass Spectroscopy (SF-ICPMS) following acid extraction, while analysis of the samples for inorganic anions was achieved by ion chromatography (IC) ( Zhang *et al.*, 2008). Total carbon was determined using a Sievers 900 Total Carbon Analyzer ( Sullivan *et al.*, 2004). Uncertainty values for all analyses are reported in the results as analytical error. Each uncertainty value is calculated as the square root of the sum of squares of the instrument and blank uncertainty components (S.D. of triplicate analyses, S.D. of triplicate blank measurements).

### Microglial *in vitro* assays

***BV-2 Cell Culture.*** PM _2.5_ slurry samples were assayed with immortalized BV-2 microglia ( RRID: CVCL_0182) ( Eun *et al.*, 2017; Gresa-Arribas *et al.*, 2012). BV-2 cells were cultured in Dulbecco’s Modified Eagle’s Medium/Ham’s F12 50/50 Mix (DMEM F12 50/50; # 11320033, Life Technologies, Carlsbad, CA) supplemented with 10% fetal bovine serum (FBS; #45000–734, VWR, Radnor, PA), 1% penicillin/streptomycin (#P4333–100ML, Sigma-Aldrich, St. Louis, MO), and 1% L-glutamine (Glutamax; #35050061, Life Technologies, Carlsbad, CA) in a humidified incubator (37 °C/5% CO _2_). For cell treatments, PM _2.5_ slurries were diluted in the same isotonic and pH-balanced culture media and applied to cells for up to 24 hours. Cell culture experiments were done in triplicate per endpoint.

***Nitrite Assay.*** Nitric oxide (NO) was assayed in BV-2 cell media by the Griess reagent ( Cheng *et al.*, 2016b; Ignarro *et al.*, 1993). BV-2 cells at 60–70% confluence in 96-well plates (2 × 10 ^6^ cells/plate) were treated with both am-PM _2.5_ and pm-PM _2.5_ at doses of 1, 5 and 20 µg/mL, 200 µL/well. At 30-minute, 60-minute and 24-hour timepoints, duplicate 50 µL aliquots of cell media were removed from each treatment well and transferred to a new 96-well plate. Within this same 96-well plate, a series of nitrite standards (50 µL/well) ranging from 0.10 to 10 µM prepared from a NaNO _2_ stock solution were added, thus allowing a standardization curve to be generated for use in determining the NO concentration in each treatment well from measured absorbance data. After transferring all aliquots, 50 µL of Griess reagent was added to each well and the plate was allowed to incubate at room temperature (21–23 °C) for 10 minutes, followed by spectrophotometric analysis at 548 nm absorbance using a SpectraMax M2 microplate reader (Molecular Devices, San Jose, CA, USA). The nitrite assay was performed in triplicate, with six data points collected at each PM _2.5_ concentration per condition.

***Quantitative Polymerase Chain Reaction (qPCR).*** The quantitative polymerase chain reaction (qPCR) assay was used to quantify upregulation of cytokines and chemokines associated with the microglial neuroinflammatory response, including IL-6, CCL2 (MCP-1), and IL-1β. BV-2 microglia were seeded in 6-well plates at 10 ^6^ cells/well and grown overnight at 37 °C/5% CO _2_, followed by treatment with aqueous am-PM _2.5_ and pm-PM _2.5_ slurries diluted to 10 μg/mL in isotonic and pH-balanced cell culture media. A control condition, consisting of pure media diluted with ultrapure water, was also prepared. After 24 hours of incubation, treated cells were trypsinized and harvested for RNA extraction. Total cell RNA was extracted using the TRIzol reagent (Invitrogen, Carlsbad, CA), and cDNA was prepared from 1 μg of RNA (RT Master Mix, BioPioneer, San Diego, CA). Specific primers for each gene were used in conjunction with the qPCR Master Mix (BioPioneer) to run real time qPCR reactions.

Genes examined by qPCR included IL-1β (forward: 5’ CTAAAGTATGGGCTGGACTG 3’; reverse: 5’ GGCTCTCTTTGAACAGAATG 3’), IL-6 (forward: 5’ TGCCTTCTTGGGACTGATGCT 3’; reverse: 5’ GCATCCATCATTTCTTTGTAT 3’), MCP-1 (forward: 5’ CCCAATGAGTAGGCTGGAGA 3’; reverse: 5’ TCTGGACCCATTCCTTCTTG 3’), and GAPDH (forward: 5’ AGACAGCCGCATCTTCTTGT 3’; reverse: 5’ CTTGCCGTGGGTAGAGTCAT 3’) (Integrated DNA Technologies, Skokie, IL). Data were normalized to GAPDH and quantified as ΔΔCt. qPCR was repeated, with 12 data points collected per treatment (am-PM _2.5_ and pm-PM _2.5_; 10 µg/mL).

***Statistical analysis.*** Results were evaluated by 2-way repeated measures ANOVA statistical analysis and Bonferroni post hoc tests using GraphPad Prism (v. 6.04) statistical software.

## Results

### Nitric Oxide (NO)

**Figure 1.**
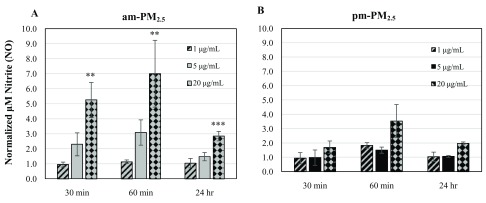
Nitric oxide (NO) induction by microglia. BV-2 microglial responses to PM _2.5_ slurries *in vitro*, assayed in culture media by the Griess reaction (control = 1.0 µM nitrite). **A.** Morning samples (am-PM _2.5_); **B.** Afternoon samples (pm-PM _2.5_). am-PM _2.5_ samples induced consistently higher NO responses for all concentrations and post-exposure timepoints. At 30 minutes post-treatment, there was a significant effect of am-PM _2.5_, as well as a significant difference between the responses to am-PM _2.5_ and pm-PM _2.5_ (overall ANOVA: p = 0.0017; am-PM _2.5_ 20 µg/mL vs. control: 5.3-fold increase, p = 0.0020; am-PM _2.5_ 20 µg/mL vs. pm-PM _2.5_ 20 µg/mL: 3.1-fold increase, p = 0.0094). There was also a significant effect of am PM _2.5_ at 60 minutes post-treatment (overall ANOVA: p = 0.010; am-PM _2.5_ 20 µg/mL vs. control: 7.0-fold increase, p = 0.0077). At 24 hours a significant effect of am-PM _2.5_ treatment was also observed (overall ANOVA: p = 0.0005; am-PM _2.5_ 20 µg/mL vs. control: 2.9-fold increase, p = 0.0007). Mean ± SE (n = 3 experiments). 2-way repeated measures ANOVA statistical analysis with Bonferroni post hoc tests: *p≤0.05, **p≤0.01, ***p≤0.001, ****p≤0.0001.

A dose-dependent NO response to PM _2.5_ treatments relative to control was observed at all timepoints (30 min., 60 min., 24 hr.), which was greater for am-PM _2.5_ than pm-PM _2.5_ exposures ( Figure 1). am-PM _2.5_ samples induced consistently higher levels of NO for all concentrations and post-exposure timepoints, with a peak effect, 7-fold greater than control (p = 0.0077), observed at 60 minutes in response to the highest am-PM _2.5_ dose of 20 µg/mL ( Figure 1A). At 30 minutes post-treatment, there was also a significant 5.3-fold increase of am-PM _2.5_ relative to control (p = 0.0020), and a significant difference between the responses to am-PM _2.5_ and pm-PM _2.5_, with am-PM _2.5_ eliciting a 3.1-fold greater NO response than pm-PM _2.5_ (p = 0.0094). There was also a significant a significant 2.9-fold increase of am-PM _2.5_ relative to control (p = 0.0007) at 24 hours post-treatment. The NO responses to pm-PM _2.5_ paralleled the effects of am-PM _2.5_ exposures, but were at least 50% smaller ( Figure 1B): the 20 µg/mL pm-PM _2.5_ treatment induced 1.7-, 3.5-, and 2.0-fold increases in NO concentration relative to control at 30 min., 60 min. and 24 hrs., respectively, but these effects were not significant. The acute effects of PM exposure seen within the first hour of exposure, at 30 and 60 minutes post-treatment, are due to direct NO induction, while the sustained effect still measurable after 24 hours indicates that there has been upregulation of the iNOS enzyme that produces NO. Thus, this overall effect is two-fold, with the increase in NO secretion due to PM _2.5_ exposure mediated by two distinct mechanisms, acute and delayed.

### Inflammatory gene responses

**Figure 2.**
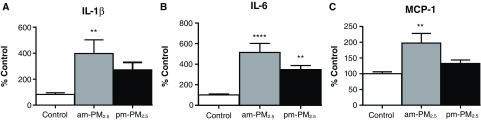
Inflammatory gene mRNA induction in microglia. After exposing BV-2 cells to 10µg/mL of morning (am-PM _2.5_) and afternoon (pm-PM _2.5_) slurries, cellular mRNA production was assessed by qPCR. Relative to control, both am-PM _2.5_ and pm-PM _2.5_ exposures increased mRNA levels of **A.** Interleukin 1β (IL-1β), **B.** Interleukin 6 (IL-6), and **C.** monocyte chemoattractant protein 1 (MCP-1). Treatment with am-PM _2.5_ induced a significant 4.8-fold increase in IL-1β expression relative to control (overall ANOVA: p = 0.0090; am-PM _2.5_: 4.8-fold increase, p = 0.0070). Both am-PM _2.5_ and pm-PM _2.5_ induced significant increases in IL-6 mRNA production (overall ANOVA: p < 0.0001; am-PM _2.5_: 5.1-fold increase, p < 0.0001; pm-PM _2.5_: 3.5-fold increase, p = 0.0046). Treatment with am-PM _2.5_ also induced a significant increase in MCP-1 mRNA production, while pm-PM _2.5_ had an effect 33% smaller than am-PM _2.5_ (overall ANOVA: p = 0.0028; am-PM _2.5_: 2.0-fold increase, p = 0.0022; am-PM _2.5_ vs. pm-PM _2.5_: p = 0.0527). Mean ± SE (n = 12). 2-way repeated measures ANOVA statistical analysis with Bonferroni post hoc tests: *p≤0.05, **p≤0.01, ***p≤0.001, ****p≤0.0001.

BV-2 cells were treated with 10 μg/mL of am-PM _2.5_ and pm-PM _2.5_ and analyzed for mRNA responses by qPCR after 24 hours incubation. The 10 μg/mL dose was chosen as below threshold for metabolic impairment based on prior studies from our group (e.g. Cheng *et al.*, 2016b; Morgan *et al.*, 2011; Woodward *et al.*, 2017b). Induction of all three cytokines was increased by both morning and afternoon PM _2.5_ samples, with more modest responses to pm-PM _2.5_ ( Figure 2). As shown in Figure 2A, treatment with am-PM _2.5_ induced a significant 4.8-fold increase in IL-1β expression relative to control (p = 0.0070). Both am-PM _2.5_ and pm-PM _2.5_ induced significant increases in IL-6 mRNA production relative to control, with am-PM _2.5_ exposure resulting in a 5.1-fold increase (p < 0.0001) and pm-PM _2.5_ resulting in a 3.5-fold increase (p = 0.0046) ( Figure 2B). Treatment with am-PM _2.5_ also induced a significant 2.0-fold increase in MCP-1 mRNA production (p = 0.0022), while pm-PM _2.5_ had a 33% smaller effect ( Figure 2C). This difference in MCP-1 mRNA production induced by am-PM _2.5_ as compared to pm-PM _2.5_ was marginally significant (p = 0.0527).

### Chemical composition of PM _2.5_ slurry samples

**Figure 3.**
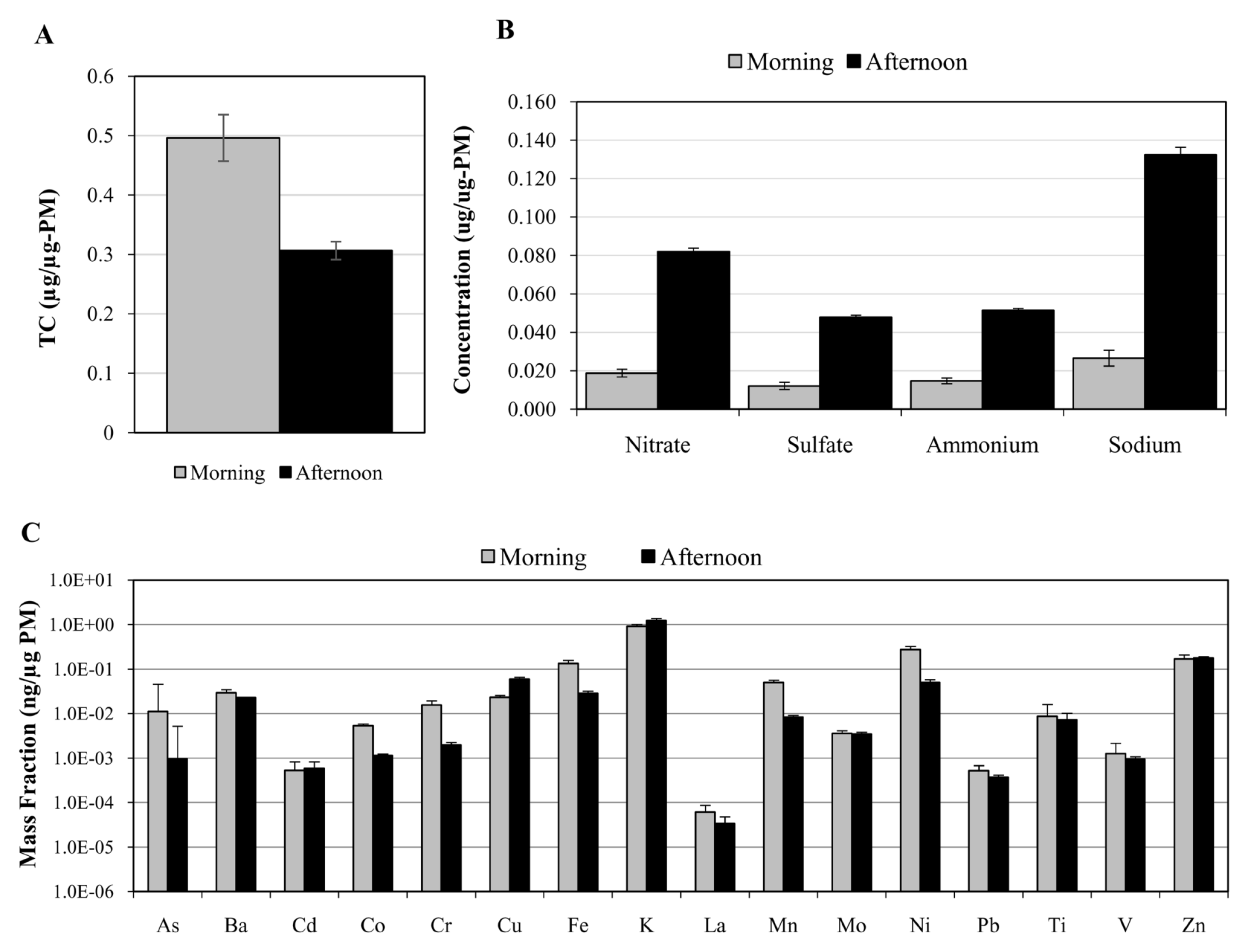
Chemical analyses. Time-integrated PM _2.5_ slurries collected during morning (6–9am) and afternoon (12–4pm) periods analyzed for **A.** Total Carbon (TC), **B.** Inorganic ions (ion chromatography), **C.** Total metals and trace elements (ICP-MS). Mean values presented are based on triplicate analysis of one sample aliquot. Error bars represent laboratory uncertainty values based on contributions of analytical error (standard deviation) and blank subtraction (standard deviation of at least three method blanks).

The am-PM _2.5_ and pm-PM _2.5_ time-integrated aqueous slurry samples were analyzed for chemical composition, including total carbon (TC), inorganic ions, and total metals and trace elements, and are presented as PM _2.5_ mass fractions in Figures 3A, 3B, and 3C, respectively. PM _2.5_ TC content decreased by 40% from morning (0.50 μg/μg-PM) to afternoon (0.31 μg/μg-PM) ( Figure 3A). Mass concentrations of inorganic secondary ions (NO _3_^-^, SO _4_^2-^, NH _4_^+^, Na ^+^) were approximately 5-fold higher in the afternoon as compared to morning slurries ( Figure 3B). For the sixteen metals and trace elements analyzed, the am-PM _2.5_ slurry contained higher mass concentrations of several measured elements as compared to the pm-PM _2.5_ slurry ( Figure 3C, note log scale; Table S1, Supplementary File 1). Arsenic, chromium, and manganese showed the largest diurnal decline, represented as am-PM _2.5_:pm-PM _2.5_ ratios: arsenic (11.6), chromium (7.9), and manganese (6.0).

The following raw data sets are provided as comma separated values (.csv) files PM_Diurnal_Variation_NO_Fig1_DATA
PM_Diurnal_Variation_qPCR_Fig2_DATA
PM_Diurnal_Variation_TC_Fig3A_DATA
PM_Diurnal_Variation_Ions_Fig3B_DATA
PM_Diurnal_Variation_Metals_Fig3C_DATA Click here for additional data file. Copyright: © 2018 Lovett C et al. 2018 Data associated with the article are available under the terms of the Creative Commons Zero "No rights reserved" data waiver (CC0 1.0 Public domain dedication).

## Discussion

Diurnal variations in urban PM _2.5_ oxidative and proinflammatory activity showed consistent decreases from morning to afternoon sampling periods in two independent *in vitro* assays using the BV-2 microglia cell line. The collection of total PM _2.5_ as an aqueous slurry was enabled by direct aerosol-into-liquid sampling that more efficiently captures water-insoluble components of ambient PM _2.5_ than traditional filter-based sampling methods used in several prior studies (e.g. Saffari *et al.*, 2015; Verma *et al.*, 2009). These slurry samples are more representative of the full range of ambient PM components and their toxicities than filter-trapped and water eluted PM. Additionally, the results of the NO assay and the qPCR assay for inflammatory gene responses extend findings from the widely used dithiothreitol (DTT) and alveolar macrophage (dichlorodihydrofluorescein, DCFH) assays for oxidative potential, which can be confounded by oxidative recycling from transition metals ( Forman & Finch, 2018). Our findings, that primary PM _2.5_ results in a greater oxidative and proinflammatory response than secondary PM _2.5_, are contrary to expectations based on prior reports that secondary, photo-oxidized PM exhibits greater oxidative activity than primary PM.

While there may be a concern that the concentrations of PM _2.5_ treatments used in the *in vitro* assays do not reflect the exact concentrations of PM _2.5_ reaching microglia in the brain following ambient exposures, these assays do serve as a useful model for how brain cells in living organisms would respond to PM _2.5_ at the given concentrations (i.e. 1, 5, 10, and 20 μg/mL). The focus of the paper was to investigate the differences between morning (primary-dominated) and afternoon (secondary-dominated) PM _2.5_, rather than to quantify actual exposure concentrations, and subsequent CNS concentrations, that would be considered harmful. We modeled these interactions between PM _2.5_ and microglia using concentrations that are below the threshold for cell death, as evaluated by the MTT assay. While there is evidence that particles can directly enter the brain through the olfactory tract ( Oberdörster *et al.*, 2004), and thus perhaps maintain higher concentrations than PM _2.5_ passing through the periphery, the concentration of particles interacting directly with brain cells via this route has not been quantified, and thus comparisons to these results could not be made.

Previous studies of diurnal variations in PM composition and oxidative activity have not been consistent and were limited in using only simple assays of oxidative potential (i.e. DTT and DCFH) on filter-captured PM. Relying solely on oxidative potential measures such as the DTT and DCFH assays provides us with only an imprecise measure of cellular oxidative and proinflammatory activity that lacks specificity. The current study improves on the experimental design of past studies by utilizing direct measures of acute oxidative stress and inflammation, including free radical production induced by PM as nitric oxide (NO) and cellular proinflammatory mRNA responses. Additionally, by using the direct aerosol-into-liquid method to collect aqueous slurries in our study, water-insoluble PM species were more efficiently captured, providing samples more representative of the full range of ambient PM components and their toxicities.

Further insight into the sources of particulate toxicity may be gleaned by the apportionment of redox properties to its water soluble and insoluble chemical components, including water-soluble and water-insoluble organic carbon (WSOC and WIOC, respectively). WSOC species are generally defined as hydrophilic, while WIOC are hydrophobic ( Turpin & Lim, 2001). Wang *et al.* (2013b) collected aqueous PM _2.5_ slurries by a similar aerosol-into-liquid sampling method, and found that increased WIOC content in PM _2.5_, relative to WSOC content, was highly correlated with redox activity on a per mass basis, indicating a greater intrinsic toxicity of WIOC as compared to WSOC. While this study was limited by its use of the DCFH assay, the greater oxidative potential associated with increased WIOC mass concentrations was attributed to organic compounds such as PAHs, as well as iron and other transition metals.

Our results indicate that morning PM _2.5_, which contains a greater proportion of water-insoluble species, may be intrinsically more toxic and induce greater cellular oxidative stress, than afternoon PM _2.5_ samples that contain a larger mass fraction of oxidized, water-soluble species that are products of photochemical reactions in the atmosphere ( Seinfeld & Pandis, 2016), including the inorganic secondary ions NO _3_^-^, SO _4_^2-^, NH _4_^+^, and Na ^+^. The mechanisms underlying the greater toxicity of primary, morning PM _2.5_ may involve non-polar WIOC components, such as PAHs, being able to more easily permeate the hydrophobic lipid-bilayer of cell membranes to trigger the formation of intracellular oxidative species and induce proinflammatory cytokine formation via an acute oxidative stress response.

Primary, traffic-derived PM _2.5_ also consists of greater concentrations of redox active and other toxic metals, as compared to the bulk of secondary PM _2.5_, which consists largely of hydrophilic products of photochemical oxidation. The metals and trace elements we found to be more prevalent in the morning slurry sample included the heavy metals vanadium, chromium, nickel, and arsenic, which are emitted by vehicles both as fuel combustion products as well as remnants of motor oil degradation ( Geller *et al.*, 2006), copper, which is associated with vehicular brake wear ( Garg *et al.*, 2000; Sanders *et al.*, 2003; Sternbeck *et al.*, 2002), and zinc, which is primarily a product of tire deterioration ( Singh *et al.*, 2002). Elevated levels of these metals in both collection periods correspond to vehicular emissions as the major source of primary particles in close proximity to the I-110 freeway. We believe the higher proportions of these metals and WIOC components in primary PM _2.5_ dominant in the morning hours, as compared to photo-oxidized secondary PM _2.5_ prevalent in the afternoon, are responsible for the diurnal variation in acute oxidative stress observed in the current study.

## Summary and conclusions

The data presented in this study demonstrate that urban PM _2.5_ collected during the morning rush hour (6–9am), when primary, traffic-derived PM emissions are dominant, induces greater oxidative and proinflammatory responses in cells as compared to PM _2.5_ collected in the afternoon (12–4pm), which contains a higher proportion of photo-oxidized, secondary PM products. Two *in vitro* assays of the cellular inflammatory response consistently demonstrated greater oxidative and proinflammatory activity due to primary (morning) PM _2.5_ exposure. We attribute this effect to the greater transition metal and water-insoluble organic carbon (WIOC) content of primary PM _2.5_, two classes of PM components that increase toxicity ( Cho *et al.*, 2005; Hu *et al.*, 2008; Li *et al.*, 2009; Shirmohammadi *et al.*, 2015; Tao *et al.*, 2003; Zhang *et al.*, 2008). Our study also improves upon previous research of diurnal variations in PM-induced oxidative stress by utilizing a unique aerosol-into-liquid PM collection system that more efficiently captures water insoluble components, thus providing complete aqueous PM samples more representative of ambient PM.

This research will ultimately help us gain a more complete understanding of the complex nature of particulate matter and how its composition and proinflammatory effects change over time due to photochemical aging in the atmosphere. The Southern California climate of Los Angeles with abundant sunshine, compounded with dense vehicular traffic, generates ubiquitous primary and secondary PM throughout the year. Identifying the health effects of these pollutants is critical as we strive to understand the underlying mechanisms of PM-induced oxidative stress, neuroinflammation and associated morbidity. Our findings may help in further elucidating the role of PM in the etiology, onset and development of widespread, chronic diseases that plague urban populations, including cancer, cardiac and respiratory distress, and neurodegenerative disorders such as Alzheimer’s disease.

## Data availability

The data referenced by this article are under copyright with the following copyright statement: Copyright: © 2018 Lovett C et al.

Data associated with the article are available under the terms of the Creative Commons Zero "No rights reserved" data waiver (CC0 1.0 Public domain dedication). 

Dataset 1: The following raw data sets are provided as comma separated values (.csv) files: 10.5256/f1000research.14836.d203329 ( Lovett *et al.*, 2018)

PM_Diurnal_Variation_NO_Fig1_DATA

PM_Diurnal_Variation_qPCR_Fig2_DATA

PM_Diurnal_Variation_TC_Fig3A_DATA

PM_Diurnal_Variation_Ions_Fig3B_DATA

PM_Diurnal_Variation_Metals_Fig3C_DATA

## Supplementary material

**Table S1.** Average concentrations and uncertainty values of total carbon, inorganic ions, metals and trace elements in ambient PM _2.5_ slurry samples collected during morning and afternoon periods.
