# Supplementary material for: Diurnal variation in the proinflammatory activity of urban fine particulate matter (PM 2.5) by in vitro assays
Source: F1000Res. 2018 Oct 1;7:596. Originally published 2018 May 15. [Version 3] doi: 10.12688/f1000research.14836.3 (PMC6171724; doi:10.12688/f1000research.14836.3)
Supplement: Supplementary file 2 [file f1000research-7-17931-s0001.tgz › 02bf92df-ec5c-4f4c-87fc-0b62e13d6955.docx]

**Table S1.** Average concentrations and uncertainty values of total carbon, inorganic ions, metals and trace elements in ambient PM_2.5_ slurry samples collected during morning and afternoon periods.

| **Compound** | **Morning** | | **Afternoon** | | **Ratio** |
| --- | --- | --- | --- | --- | --- |
|  | Average Conc. | Uncertainty | Average Conc. | Uncertainty | (morning/ afternoon) |
| ***Metals/Trace Elements*** | (ng/mg-PM) | (ng/mg-PM) | (ng/mg-PM) | (ng/mg-PM) |  |
| As | 0.011 | 0.035 | 0.0010 | 0.0042 | 11.6 |
| Ba | 0.030 | 0.0051 | 0.023 | 0.00055 | 1.30 |
| Cd | 0.00053 | 0.00029 | 0.00058 | 0.00024 | 0.91 |
| Co | 0.0053 | 0.00054 | 0.0011 | 0.00011 | 4.71 |
| Cr | 0.016 | 0.0036 | 0.0020 | 0.00029 | 7.91 |
| Cu | 0.023 | 0.0025 | 0.059 | 0.006 | 0.39 |
| Fe | 0.13 | 0.024 | 0.028 | 0.0033 | 4.69 |
| K | 0.92 | 0.089 | 1.24 | 0.13 | 0.74 |
| La | 0.000061 | 0.000025 | 0.000034 | 0.000014 | 1.82 |
| Mn | 0.050 | 0.0062 | 0.0083 | 0.00076 | 6.02 |
| Mo | 0.0036 | 0.00052 | 0.0035 | 0.00032 | 1.03 |
| Ni | 0.27 | 0.050 | 0.050 | 0.0083 | 5.46 |
| Pb | 0.00052 | 0.00016 | 0.00037 | 0.000043 | 1.41 |
| Ti | 0.0087 | 0.0073 | 0.0073 | 0.0029 | 1.20 |
| V | 0.0013 | 0.00089 | 0.00094 | 0.00014 | 1.33 |
| Zn | 0.17 | 0.039 | 0.18 | 0.015 | 0.97 |
| ***Inorganic Ions*** | (µg/µg-PM) | (µg/µg-PM) | (µg/µg-PM) | (µg/µg-PM) |  |
| NO_3_^-^ | 0.019 | 0.0020 | 0.082 | 0.0017 | 0.23 |
| SO_4_^2-^ | 0.012 | 0.0018 | 0.048 | 0.0009 | 0.25 |
| NH_4_^+^ | 0.015 | 0.0015 | 0.051 | 0.0010 | 0.29 |
| Na^+^ | 0.027 | 0.0041 | 0.13 | 0.0038 | 0.20 |
| ***Total Carbon*** | 0.50 | 0.039 | 0.31 | 0.015 | 1.62 |
|  |  |  |  |  |  |
