# Supplementary material for: Diurnal variation in the proinflammatory activity of urban fine particulate matter (PM 2.5) by in vitro assays
Source: F1000Res. 2018 Oct 1;7:596. Originally published 2018 May 15. [Version 3] doi: 10.12688/f1000research.14836.3 (PMC6171724; doi:10.12688/f1000research.14836.3)
Supplement: Peer review history

# Peer review: Referee response for version 3

**DOI:** 10.5256/f1000research.17931.r38889

**Published:** 2018-10-01

**Authors:** Kent E. Pinkerton

**Affiliations:** 1 Center for Health and the Environment, University of California, Davis, Davis, CA, USA

The authors have adequately addressed all remaining concerns presented by the reviewers. This manuscript has full approval for indexing.

I have read this submission. I believe that I have an appropriate level of expertise to confirm that it is of an acceptable scientific standard.

# Peer review: Referee response for version 2

**DOI:** 10.5256/f1000research.16924.r36102

**Published:** 2018-09-03

**Authors:** Kent E. Pinkerton

**Affiliations:** 1 Center for Health and the Environment, University of California, Davis, Davis, CA, USA

The research article by Lovett and colleagues, Diurnal variation in the proinflammatory activity of urban fine particulate matter (PM _2.5_) by *in vitro* assays”, is a fascinating and provocative article, however, not without some controversy in its interpretation.

 The authors state ambient PM _2.5 _undergoes diurnal changes in chemical composition due to photochemical oxidation. In this study the authors examined the relationships between oxidative activity and inflammatory responses associated with these diurnal chemical changes. Because secondary PM contains a higher fraction of oxidized PM species, the authors hypothesize PM _2.5_ collected during afternoon hours induce a greater inflammatory response than primary, morning PM _2.5_.

 The methods used for PM collections methods are highly appropriate and well executed. The in vitro biological methods implemented with immortalized microglia cells are logical and clearly described. The authors state microglia were used for *in vitro *assays of oxidative and inflammatory responses to PM2.5 exposures because of their increasingly recognized role in environmental neurotoxicology. These are all correct statements. The difficulty comes in the interpretation of the findings.

 Specific Comments: 

1. It is unclear how the dose of PM delivered to microglia in vitro would compare to the in vivo setting where PM and/or oxidation reaction products would need to be transported either through the olfactory epithelium of the nasal cavity and/or via the blood passing through the lungs. The authors need to provide further clarification as to the credibility of using microglia cells in culture to mimic neurotoxicology. The authors need to clarify these concerns.
2. A five week sampling campaign was conducted by the authors during late summer (August and September) of 2016, ensuring maximum UV sunlight exposure to enhance photochemical oxidation reactions seems reasonable. However, how was the stability of PM samples maintained over such a prolonged period of sampling of five weeks? How stable are photochemical oxidation reactions?
3. PM _2.5_ slurry samples were assayed with immortalized BV-2 microglia. It is unclear in the text what was the concentration of PM _2.5_ slurry samples used for these assays. Please state in the text.
4. Based on Figure 1, were 1, 5 and 20 um/ul of PM _2.5_ used? How do these doses compare to the concentration of PM _2.5_ actually reaching microglia cells in vivo? 
5. Nitric oxide (NO) was assayed in BV-2 cell media by the Griess reagent at 30, 60 minute and 24 hour timepoints. A NaNO _2_ stock solution used to allow the creation of a standardization curve to determine the NO concentration in each treatment well from measured absorbance data. The authors observed a dose-dependent NO response to PM _2.5_ treatments relative to control at all timepoints (30 min., 60 min., 24 hr.). This assay demonstrated greater effects for morning PM _2.5_ than for afternoon PM _2.5_. This assay is quite remarkable, meritorious and clearly illustrated. The interpretation of these findings need to be clearly stated.
6. Summary and Conclusion. The first portion of this section is nicely written to state “urban PM _2.5_ collected during the morning rush hour (6–9am), when primary, traffic-derived PM emissions are dominant, induces greater oxidative and proinflammatory responses in cells as compared to PM _2.5_ collected in the afternoon (12–4pm), which contains a higher proportion of photo-oxidized, secondary PM products”. It is unclear whether the authors have provided conclusive evidence of these diurnal differences in terms of PM chemistry. Please clarify.
7. Summary and Conclusion. The authors state, “Two *in vitro *assays of the cellular inflammatory response consistently demonstrated greater oxidative and proinflammatory activity due to primary (morning) PM _2.5_ exposure. We attribute this effect to the greater transition metal and water-insoluble organic carbon (WIOC) content of primary PM _2.5_, two classes of PM components that increase toxicity. Again, how conclusive is the chemical analysis of PM from these two periods for PM _2.5_ collected over a period of five weeks?
8. Summary and Conclusion. The authors state, “This research will ultimately help us gain a more complete understanding of the complex nature of particulate matter and how its composition and proinflammatory effects change over time due to photochemical aging in the atmosphere. The Southern California climate of Los Angeles with abundant sunshine, compounded with dense vehicular traffic, generates ubiquitous primary and secondary PM throughout the year. Identifying the health effects of these pollutants is critical as we strive to understand the underlying mechanisms of PM-induced oxidative stress, neuroinflammation and associated morbidity”. This is a laudatory conclusion made by the authors using in vitro cells that may or may not represent in vivo conditions of cell response and/or PM dose delivered to the nervous system. The authors need to clearly state the advantages of their study, along with the limitations for interpretation and extrapolation to actual in vivo conditions of exposure.
9. Summary and Conclusion. The authors state, “Our findings may help in further elucidating the role of PM in the etiology, onset and development of widespread, chronic diseases that plague urban populations, including cancer, cardiac and respiratory distress, and neurodegenerative disorders such as Alzheimer’s disease”. Should the authors acknowledge the limitations of the methods used in this study, this concluding statement is reasonable.

I have read this submission. I believe that I have an appropriate level of expertise to confirm that it is of an acceptable scientific standard, however I have significant reservations, as outlined above.

# Response

**Published:** 2018-09-24

**Authors:** Christopher Lovett

**Affiliations:** University of Southern California, USA

Authors’ responses to specific comments of Dr. Kent Pinkerton:

Comment 1: *It is unclear how the dose of PM delivered to microglia *in vitro *would compare to the *in vivo *setting where PM and/or oxidation reaction products would need to be transported either through the olfactory epithelium of the nasal cavity and/or via the blood passing through the lungs. The authors need to provide further clarification as to the credibility of using microglia cells in culture to mimic neurotoxicology. The authors need to clarify these concerns.*

**Authors’ Response:** While we acknowledge the limitations of *in vitro* assays, we believe the reliability of the *in vitro* assays in characterizing neurotoxicology, as compared to *in vivo* experiments, has been adequately documented in the several studies cited in the paper (e.g. Morgan, et al., 2011; Davis et al., 2013; Cheng et al., 2016b; Woodward et al., 2017b). Additionally, the specific use of cultured BV-2 cells (microglia) successfully in *in vitro* assays of neurotoxicity has also been demonstrated, as cited in the paper (e.g. Gresa-Arribas et al., 2012; Eun et al., 2017).

Comment 2: *A five-week sampling campaign was conducted by the authors during late summer (August and September) of 2016, ensuring maximum UV sunlight exposure to enhance photochemical oxidation reactions seems reasonable. However, how was the stability of PM samples maintained over such a prolonged period of sampling of five weeks? How stable are photochemical oxidation reactions?*

**Authors’ Response:** The PM samples were collected daily over the course of 5 weeks using the direct aerosol-into-liquid sampling system (Wang et al., 2013a). At the end of each sampling period, morning and afternoon, each daily aqueous sample was added to a total sample collection bottle kept under refrigeration. Once collected and refrigerated, these cumulative aqueous samples were used in the *in vitro* experiments discussed in the paper. It is possible that changes in PM composition might occur during sampling, but this is the advantage of using the direct aerosol-into-liquid system as opposed to filter-based sampling. PM is collected directly into an aqueous suspension and does not have to undergo an aqueous extraction and re-suspension process, which thereby significantly reduces the possibility of any artifact formation. The benefits of this collection method compared to conventional filter samplers are discussed in several prior publications (Zhao et al., 2005; Wang et al., 2013b; Saarikoski et al., 2014).

**Added text (Methods: Particulate sample collection):**

 “At the end of each morning and afternoon daily sampling period, each aqueous slurry sample was added to its corresponding total sample collection bottle that was kept refrigerated. At the end of the 5-week sampling period, these continuously refrigerated, cumulative aqueous slurry samples were then used in the *in vitro* assays. While it is possible that changes in PM composition might occur during sampling, the advantage of using the direct aerosol-into-liquid system is that PM is collected directly into an aqueous suspension and does not undergo an aqueous extraction and re-suspension process, thereby significantly reducing the possibility of any artifact formation. The benefits of this collection method compared to conventional filter sampling systems has been discussed extensively in the literature (e.g. Zhao et al., 2005; Wang et al., 2013b; Saarikoski et al., 2014).”

**Additional References (added to paper):**

 Zhao, Y., Bein, K. J., Wexler, A. S., Misra, C., Fine, P. M., & Sioutas, C. (2005). Field evaluation of the versatile aerosol concentration enrichment system (VACES) particle concentrator coupled to the rapid single‐particle mass spectrometer (RSMS‐3). *Journal of Geophysical Research: Atmospheres*, *110*(D07S02).

 Saarikoski, S., Carbone, S., Cubison, M. J., Hillamo, R., Keronen, P., Sioutas, C., Worsnop, D. R., & Jimenez, J. L. (2014). Evaluation of the performance of a particle concentrator for online instrumentation. *Atmospheric Measurement Techniques*, *7*(7), 2121-2135.

Comment 3: *PM _2.5_ slurry samples were assayed with immortalized BV-2 microglia. It is unclear in the text what was the concentration of PM _2.5_ slurry samples used for these assays. Please state in the text.*

**Authors’ Response: **In the Methods section, the concentrations of PM _2.5_ slurry samples used in each assay are stated, including the nitrite assay (Methods section: Nitrite Assay), which used 1, 5 and 20 µg/mL, and the qPCR assay (Methods section: Quantitative Polymerase Chain Reaction (qPCR)), which used 10 µg/mL.

Comment 4: *Based on Figure 1, were 1, 5 and 20 μg/ml of PM _2.5_ used? How do these doses compare to the concentration of PM _2.5_ actually reaching microglia cells *in vivo *?*

**Authors’ Response: **In the Nitrite Assay (Figure 1), concentrations of 1, 5 and 20 µg/mL were used.  In the qPCR assay (Figure 2), the PM _2.5­_ concentration used was 10 µg/mL. We do not know the concentration of PM _2.5_ reaching microglia in the brain following ambient exposure, however the *in vitro* assays were used as a model for how brain cells respond to PM _2.5_ at the given concentrations. The focus of the paper was to investigate the differences between morning (primary-dominated) and afternoon (secondary-dominated) PM _2.5_, rather than quantifying the actual exposure concentrations, and resulting CNS concentrations, that would be considered harmful. We modeled these interactions between PM _2.5_ and microglia using concentrations that are below the threshold for cell death, as evaluated by the MTT assay. While there is evidence that particles can directly enter the brain through the olfactory tract (Oberdörster et al., 2004), the concentration of particles interacting directly with brain cells via this route has not been quantified, and thus comparisons could not be made.

**Added text (Discussion section: paragraph 2):**

 “While there may be a concern that the concentrations of PM _2.5_ treatments used in the *in vitro* assays do not reflect the exact concentrations of PM _2.5_ reaching microglia in the brain following ambient exposures, these assays do serve as a useful model for how brain cells in living organisms would respond to PM _2.5_ at the given concentrations (i.e. 1, 5, 10, and 20 μg/mL). The focus of the paper was to investigate the differences between morning (primary-dominated) and afternoon (secondary-dominated) PM _2.5_, rather than to quantify actual exposure concentrations, and subsequent CNS concentrations, that would be considered harmful. We modeled these interactions between PM _2.5_ and microglia using concentrations that are below the threshold for cell death, as evaluated by the MTT assay. While there is evidence that particles can directly enter the brain through the olfactory tract (Oberdörster et al., 2004), and thus perhaps maintain higher concentrations than PM _2.5_ passing through the periphery, the concentration of particles interacting directly with brain cells via this route has not been quantified, and thus comparisons to these results could not be made.”

**Additional Reference (added to paper):**

 Oberdörster, G., Sharp, Z., Atudorei, V., Elder, A., Gelein, R., Kreyling, W., & Cox, C. (2004). Translocation of inhaled ultrafine particles to the brain. *Inhalation Toxicology*, *16*(6-7), 437-445.

Comment 5: *Nitric oxide (NO) was assayed in BV-2 cell media by the Griess reagent at 30, 60 minute and 24 hour timepoints. A NaNO _2_ stock solution used to allow the creation of a standardization curve to determine the NO concentration in each treatment well from measured absorbance data. The authors observed a dose-dependent NO response to PM _2.5_ treatments relative to control at all timepoints (30 min., 60 min., 24 hr.). This assay demonstrated greater effects for morning PM _2.5_ than for afternoon PM _2.5_. This assay is quite remarkable, meritorious and clearly illustrated. The interpretation of these findings need to be clearly stated.*

**Authors’ Response:** To clarify these findings, we offer the flowing additional text:

**Added text (Results: Nitric Oxide (NO)):**

 “The acute effects of PM exposure seen within the first hour of exposure, at 30 and 60 minutes post-treatment, are due to direct NO induction, while the sustained effect still measurable after 24 hours indicates that there has been upregulation of the iNOS enzyme that produces NO. Thus, this overall effect is two-fold, with the increase in NO secretion due to PM _2.5_ exposure mediated by two distinct mechanisms, acute and delayed.”

Comment 6: *Summary and Conclusion. The first portion of this section is nicely written to state “urban PM _2.5_ collected during the morning rush hour (6-9am), when primary, traffic-derived PM emissions are dominant, induces greater oxidative and proinflammatory responses in cells as compared to PM _2.5_ collected in the afternoon (12-4pm), which contains a higher proportion of photo-oxidized, secondary PM products.” It is unclear whether the authors have provided conclusive evidence of these diurnal differences in terms of PM chemistry. Please clarify.*

**Authors’ Response:** It is well-known that over the course of daytime sunlight exposure, photochemical reactions occur, and secondary PM components, largely products of primary emissions oxidation, are formed such that secondary PM has a different chemical composition than primary PM. This is discussed in several places in the text, and relevant studies were cited (e.g. Forstner et al., 1997; Grosjean & Seinfeld, 1989; Seinfeld & Pandis, 2016). That the observed diurnal variations in cellular oxidative stress, as measured by two distinct *in vitro* assays, are related to higher concentrations of PM components associated with primary PM (i.e. transition metals and WIOC) was also discussed, and several studies were cited in support of this conclusion (e.g. Tao et al., 2003; Li et al., 2009; Cho et al., 2005; Zhang et al., 2008; Hu et al., 2008; Shirmohammadi et al., 2015).

Comment 7: *Summary and Conclusion. The authors state, “Two* in vitro *assays of the cellular inflammatory response consistently demonstrated greater oxidative and proinflammatory activity due to primary (morning) PM _2.5_ exposure. We attribute this effect to the greater transition metal and water-insoluble organic carbon (WIOC) content of primary PM _2.5_, two classes of PM components that increase toxicity.” Again, how conclusive is the chemical analysis of PM from these two periods for PM _2.5_ collected over a period of five weeks?*

**Authors’ Response: **We feel that we have provided ample and solid evidence of the compositional differences between these two sets of samples (am-PM _2.5_ and pm-PM _2.5_). We respectfully disagree that we have not provided clear evidence of these compositional differences of the two sets of samples based on the chemical analyses, specifically regarding both their organic carbon content as well as the toxic trace elements and metals components, as can be seen in Figure 3, as well as in the supplemental chemical analyses data files. If the concern is the lengthy time-integrated sample collection period of 5 weeks leading to sample instability or degradation, the aerosol-into-liquid samples are exceptionally stable given the removal of a filter-extraction step in the analysis process, particularly when kept refrigerated, as discussed above in our response to comment 2.

Comment 8: *Summary and Conclusion. The authors state, “This research will ultimately help us gain a more complete understanding of the complex nature of particulate matter and how its composition and proinflammatory effects change over time due to photochemical aging in the atmosphere. The Southern California climate of Los Angeles with abundant sunshine, compounded with dense vehicular traffic, generates ubiquitous primary and secondary PM throughout the year. Identifying the health effects of these pollutants is critical as we strive to understand the underlying mechanisms of PM-induced oxidative stress, neuroinflammation and associated morbidity”. This is a laudatory conclusion made by the authors using *in vitro *cells that may or may not represent *in vivo *conditions of cell response and/or PM dose delivered to the nervous system. The authors need to clearly state the advantages of their study, along with the limitations for interpretation and extrapolation to actual *in vivo *conditions of exposure.*

**Authors’ Response: **One methodological advantage of this study, as stated in the text, is the use of the direct aerosol-into-liquid sampler, which allows us to capture more water-insoluble PM species that may not be eluted during water extraction of filters. Another advantage of this study is that we use two separate *in vitro* assays of proinflammatory biomarkers (the nitrite assay and qPCR). Additionally, while there are limitations in extrapolating *in vitro* findings to processes occurring in living organisms, the focus of this study was to compare the relative proinflammatory effects of direct cellular exposure to morning and afternoon PM _2.5_ rather than to precisely quantify these effects *in vivo*. Additionally, studies of direct interactions between PM _2.5_ and microglia *in vivo* have not been conducted for comparison with our (and numerous other) *in vitro* studies.

Comment 9: *Summary and Conclusion. The authors state, “Our findings may help in further elucidating the role of PM in the etiology, onset and development of widespread, chronic diseases that plague urban populations, including cancer, cardiac and respiratory distress, and neurodegenerative disorders such as Alzheimer’s disease”. Should the authors acknowledge the limitations of the methods used in this study, this concluding statement is reasonable.*

**Authors’ Response: **Regarding the limitations of methods used in this study, the responses to comments and additional text incorporated into the revised manuscript, as detailed in the responses to comments above, should provide sufficient to address these concerns.

# Peer review: Referee response for version 2

**DOI:** 10.5256/f1000research.16924.r35515

**Published:** 2018-07-30

**Authors:** Ning Li

**Affiliations:** 1 Department of Pathobiology and Diagnostic Investigation, College of Veterinary Medicine (CVM), Michigan State University, East Lansing, MI, USA

**General comments**

 This study examined the relationship between diurnal changes in the chemical properties and the pro-oxidant and pro-inflammatory activities of PM2.5 collected in Los Angeles area. The hypothesis was that compared to primary, morning PM (am-PM), secondary PM in the afternoon (pm-PM) would induce a stronger inflammatory response in microglial cells. PM were collected using an aerosol-into-liquid collection system. Cellular endpoints included nitric oxide induction and the expression of IL-1b, IL-6 and MCP-1 genes. Characterization of PM included analyses of metal and trace elements, total carbon and inorganic ions. The findings were contrary to the authors’ expectation. Morning PM had stronger effects in inducing NO production and up-regulating IL-1b, IL-6 and MCP-1 gene expression than pm-PM. It was concluded that the diurnal differences between am-PM and pm-PM may be caused by the greater content of transition metals and water-insoluble organic carbon of am-PM (primary PM). This work has two strengths: 1) diurnal changes in the chemical properties and adverse health effects of ambient PM have not been well studied and 2) the use of aerosol-into-liquid collection system reduces the loss of PM components. 

**Specific comments**

1.  Fig. 1. The highest PM concentration was 20 μg/ml. The authors indicated that “The 10 μg/ml dose was chosen as below threshold for metabolic impairment based on prior studies from our group”. Was metabolic impairment assessed at 20 μg/ml? If yes, was there any cellular injury?
2.  Were endotoxin levels in these PM samples measured? Was there any difference between am-PM and pm-PM? 
3. The authors explained the rationale for not using DTT and DCF-DA assays. What was the rationale for selecting NO instead of other indicators (e.g., HO-1 or GSH/GSSG) to assess oxidative stress?
4.   Fig. 2. What are the reasons that there is no * above pm-PM2.5 in Figures 2A (IL-1b) and 2C (MCP-1)? Were pm-PM2.5-induced increases of IL-1b and MCP-1 significantly different from respective control? What is the p-value of am-PM2.5 vs. pm-PM2.5 in Fig. 2A?  
5.   Fig. 3A, 3B and 3C (As, Co, Cr, Fe, Mn and Ni). Are the differences between am-PM and pm-PM statistically significant?

I have read this submission. I believe that I have an appropriate level of expertise to confirm that it is of an acceptable scientific standard.

# Response

**Published:** 2018-07-31

**Authors:** Christopher Lovett

**Affiliations:** University of Southern California, USA

Authors’ responses to specific comments of Dr. Ning Li:

1. *Fig. 1. The highest PM concentration was 20 μg/ml. The authors indicated that “The 10 μg/ml dose was chosen as below threshold for metabolic impairment based on prior studies from our group.” Was metabolic impairment assessed at 20 μg/ml? If yes, was there any cellular injury?*

**Response:** The MTT assay was conducted with BV-2 cells and included four doses (1, 5, 10, 20 μg/mL) of PM2.5 slurry samples, followed by 24 hours incubation time. Significant reductions in mitochondrial activity occurred only at the highest PM2.5 dose, 20 μg/mL, in both am-PM2.5 and pm-PM2.5 samples. However, this activity was still above the 50% threshold. Additionally, given strong cell responses at 20 μg/mL for NO and cytokines, we infer that cells were viable at the highest dose.

2. *Were endotoxin levels in these PM samples measured? Was there any difference between am-PM and pm-PM?*

**Response:** Prior studies using the Limulus assay did not detect endotoxin (Woodward et al., 2017) in ambient PM samples collected at the same central Los Angeles location. Additionally, all sample collection and measurement equipment was routinely sanitized with 70% ethanol solution prior to use each week, per our strict laboratory hygiene protocols.

3. *The authors explained the rationale for not using DTT and DCF-DA assays. What was the rationale for selecting NO instead of other indicators (e.g., HO-1 or GSH/GSSG) to assess oxidative stress?*

**Response:** Nitric oxide (NO) secretion, measured by the Griess assay, was chosen as a biomarker of the oxidative stress response based on its reliability as an index of PM-induced oxidative stress. Our research group has previously used this measure in several published studies of nPM exposure *in vitro* (e.g. Davis et al., 2013; Cheng et al., 2016).

4. Fig. 2. What are the reasons that there is no * above pm-PM2.5 in Figures 2A (IL-1b) and 2C (MCP-1)? Were pm-PM2.5-induced increases of IL-1b and MCP-1 significantly different from respective control? What is p-value of am-PM2.5 vs. pm-PM2.5 in Fig. 2A? 

**Response:** In Figure 2A (IL-1β), the response to pm-PM2.5 was 3.3-fold above control, with marginal significance (p = 0.14). The IL-1β response to am-PM2.5 was 1.5-fold above the pm-PM2.5 response (not significant, p = 0.41). In Figure 2C (MCP-1), the response to pm-PM2.5 was 1.3-fold above control (not significant, p = 0.44).

5. *Figs. 3A, 3B and 3C (As, Co, Cr, Fe, Mn and Ni). Are the differences between am-PM and pm-PM statistically significant?*

**Response: **The chemical data presented in Figures 3A, 3B, and 3C (total carbon, inorganic ions, total metals/trace elements) include error bars representing laboratory measurement uncertainty. However, because only one time-integrated slurry sample (collected over several hours, from either 6-9am or 12-4pm, during each weekday for 5 weeks) was analyzed in each condition (n = 1), it was not possible to do an ANOVA to determine the statistical significance of differences in concentrations of various chemical species between the am-PM2.5 and pm-PM2.5 samples.
